# Traversing the valley of glycemic control despair

**DOI:** 10.1186/s13054-017-1824-9

**Published:** 2017-09-07

**Authors:** J. Geoffrey Chase, Jennifer L. Dickson

**Affiliations:** Department of Mechanical Engineering, Centre for Bio-Engineering, University of Canterbury, Private Bag 4800, Christchurch, New Zealand

**Keywords:** Glycemic control, Intensive care, Critical care, Repeatable, Clinical trial, Change, Replicable, Quality, LOGIC

The debate on glycemic control (GC) in critical care nears the 16-year mark since the initial landmark study. In this journal, Mesotten et al. [1], from the Leuven group of the original study, present the results of the LOGIC-2 multicenter trial of model-based GC versus standard nurse GC.

In 1979 Kelley and Conner [2] published the “*Emotional Cycle of Change*” defining five emotional phases involved in any major change: 1) uninformed optimism; 2) informed pessimism; 3) the valley of despair—where many “opt out”; 4) informed optimism; and eventually 5) completion and success.

We have certainly seen uninformed optimism at initial results [3], followed by many unsuccessful attempts to repeat them and the rise of (increasingly) informed pessimism [4–6]. The resulting confusion in the field led to rival camps of “believers” agreeing to disagree [7], and the valley of despair as many clinicians found GC unnecessary [8], despite strong associations between GC performance, such as time in band and reduced hypoglycemia, and clinical outcomes [9].

The results of the study by Mesotten et al, in the context of other very recent results, suggest GC has made it to step 4—informed optimism.

Very recent analysis in this journal suggests the association between mortality and glycemic levels, safety, and variability is a function of the quality of GC and not of patient condition or outcome, indicating GC can play a major role in patient outcomes [10]. Other results in the journal have indicated achieving outcome benefits requires essentially all patients receive safe, effective GC [11]. Hence, we can begin to conclude that GC is important, yet very difficult, and thus how it is implemented may matter as much as whether it is implemented.

In light of these points, the multicenter LOGIC study [1] offers two key insights into the main needs and the basis of future success in GC in critical care … thus taking us to informed optimism.

First is the need for repeatability across patients, best defined as the need for patient-specific “*one method fits all*” solutions, where many studies fail to achieve safe, effective control for (essentially) all patients [12]. Second, and most critical, is the need for repeatability across units, where many prior trials have failed at the hurdle of variable performance within, but especially across, units. The results by Mesotten et al. in [1] support both these needs.

## Repeatability across patients

Humans are horribly variable [13], and perhaps none more so than glycemically dysregulated critical care patients. Patient-specific responses to insulin and nutrition interventions can differ substantially over just a few hours between measurements [10, 14]. The use of model-based and model predictive control in [1] provides tight, safe, and effective performance matching the well-known and acclaimed nursing control in the Leuven center.

The key element of the protocol used in the LOGIC study is the ability to adapt care to changing, patient-specific condition using advanced modeling and mathematics [12]. These models, methods, and associated virtual patients offer opportunities to make care consistent to patient condition, rather than consistent to measured patient responses. This subtle difference provides a level of adaptability and specificity that is not consistently possible with most, if not all, clinical protocols, where Leuven is often the exception proving the rule. The result is highly replicable control and consistency per-patient in control across each individual critical care unit in this study.

## Repeatability across units

Hyperglycemic humans, as well as clinical practice and culture, are also horribly variable across critical care units. A key failing in many of the larger, multicenter randomized trials of GC was the inability to get consistent quality control in every unit [4–6]. Thus, patients received variable safety and performance in GC, and it became hard to obtain a significant change in patient outcomes. Mesotten et al. show very consistent GC across three different intensive care units, similar tightness to target glycemia (median blood glucose within 4 mg/dL), and similar variability (standard deviation of 9–10 mg/dL) for two of three units.

This level of consistency across units has only been shown one other time, to the best of the authors’ knowledge, with almost identical GC across two units with very different patient cohorts and clinical practice cultures [15]. This study also used patient-specific, model-predictive GC, via the STAR protocol. Thus, very close or identical replicability across intensive care units has only been achieved with model-based GC protocols—an indication of the difficulty of achieving replicable GC for both patients and across units.

Most importantly, the ability to achieve replicable results across units with different clinical approaches, patients, and cultures provides much greater confidence that a large randomized trial would provide the required quality control to all patients. In the authors’ opinion, the inability to accomplish this outcome and the confusion engendered by this inability has delayed acceptance of GC and its potential to improve outcomes far more so than any other factor.

## A strong opinion in closing

In short, this editorial opinion strongly recommends that any future GC trial be first required to show such replicability of safety, performance, and workload in standard care before commencement. We would go further and state that given the need to achieve safe, effective control for nearly all patients to ensure outcomes might be seen, the failure to do so would impose significant burden on patients and clinicians for no potential outcome.

All new protocols, treatments, and technologies involve change. The bigger the change, often the greater the resistance and difficulty to implement. Thus, as GC moves, we believe, to informed optimism, the results by Mesotten et al. and other recent results show that completion and success is achievable, but only through replicability, across all patients, and across all units.

Our goals now should be to ask what technologies, models, and other practices will best let us achieve these goals, and how to disseminate their uptake more rapidly?

## Abbreviations

GC: Glucose control and/or glycemic control
LOGIC: Leuven Online Glucose Insulin Calculator
STAR: Stochastic TARgeted
